# Supplementary material for: Vaccine imprinting drives increased SARS-CoV-2 variant infection in children
Source: bioRxiv. 2026 Aug 13:2026.08.12.739589. Preprint. [Version 1] doi: 10.64898/2026.08.12.739589 (PMC13640708; doi:10.64898/2026.08.12.739589)
Supplement: Supplementary file 1 [file media-1.pdf]

Supplementary Methods

Logistic regression – XFG and BA.3.2 sequence data

Let $I_i(t)$ be the density of hosts infected by the strain (or variant) i of the pathogen at the current time t. The total density of infected hosts is then $I(t) = \sum_i I_i(t)$. Define $r_i(t)$, the growth rate of hosts infected by pathogen variant i. The temporal dynamics of $I_i(t)$ is given by the following ordinary differential equation:

$$\dot{I}_i(t) = r_i(t) I_i(t), \tag{1}$$

and the temporal dynamics of $I(t)$ by:

$$\dot{I}(t) = \sum_i r_i(t) I_i(t) = \underbrace{\sum_i q_i(t) r_i(t)}_{\bar{r}(t)} I(t), \tag{2}$$

where $q_i(t) = I_i(t)/I(t)$ represents the frequency of hosts infected by the variant i among all infected hosts.

We are interested in tracking the frequency q_i:

$$\begin{aligned} \dot{q}_i(t) &= \frac{\dot{I}_i(t)}{I(t)} - q_i \frac{\dot{I}(t)}{I(t)} \\ &= q_i(t)\left(1 - q_i(t)\right) \underbrace{\left(r_i(t) - \frac{\sum_{j \neq i} q_j(t) r_j(t)}{\sum_{j \neq i} q_j(t)} \right)}_{\mathcal{S}_i(t)}, \end{aligned} \tag{3}$$

where we define $\mathcal{S}_i$ as the selection coefficient of variant i relative to the background population, that is, the relative growth advantage (or disadvantage) of variant i against all competing variants. Specifically, this coefficient measures the rate of change of the variant frequency on the logit scale – i.e., the log odds $\text{logit}(q_i/(1 - q_i))$ –, and provides a relevant measure for the speed of pathogen adaptation:

$$\frac{\text{d logit}\left(q_i(t)\right)}{\text{d}t} = \mathcal{S}_i(t). \tag{4}$$

When $\mathcal{S}_i(t)$ is constant (or approximately constant), the variant frequency follows a logistic growth model: $\text{logit}\left(q_i(t)\right) = \text{logit}\left(q_i(0)\right) + \mathcal{S}_i \times t$.

We collected patient status metadata of GISAID SARS-CoV-2 sequences from New York and New Jersey US states (corresponding to the Health and Human Services (HHS) region 2, excluding Puerto Rico and the Virgin Islands) between 2025-01-05 to 2026-07-04. This region was chosen because it has a relatively large number of sequences compared to other locations, with good age coverage for those sequences. We consider all lineages associated in GISAID to Variant Under Monitoring (VUM) XFG + XFG.* as the XFG lineage, and all sequences associated to VUM BA.3.2 + BA.3.2.* as the BA.3.2 lineage.

We fit a linear generalized model (logistic regression) to sequence data. Let $Y_k(t) = 1$ if the kth sequence belongs to the variant i, and 0 otherwise, such that:

$$Y_k(t) \sim \text{Bernoulli}\left(q_i(t)\right). \tag{5}$$

We thus have:

$$\Pr\left[Y_k(t) = 1\right] = q_i(t), \tag{6}$$

assuming:

$$\text{logit}\left(q_i(t)\right) = \beta_0 + \beta_1 \times t, \tag{7}$$

with β_0, the intercept and β_1, the slope. The (relative) selection coefficient, $\mathcal{S}_i$, is thus estimated by the slope β_1. To minimize the influence of demographic stochasticity during the early phase of variant emergence, we initiate the logistic regression from the first day of the first week in which the variant frequency reaches 4%. We then restrict the fit of the model over the period during which the logit-transformed variant frequency is approximately linear. The final five weeks of data were excluded to account for delays in sequence reporting and backfilling.

We also fit a logistic regression to sequence data stratified by age groups:

$$\text{logit}\left(q_{i,a}(t)\right) = \beta_0 + \delta_a + \left(\beta_1 + \gamma_a\right) \times t, \tag{8}$$

with δ_a, the difference in intercept for age group a relative to the reference age group, and γ_a, the difference in slope. Here, the selection coefficient in age group a, $\mathcal{S}_{i,a}$, is estimated by the slope $(\beta_1 + \gamma_a)$. Here, we consider two age groups: individuals aged 0–10 years and individuals aged 11 years and older.

Table S1. Cohort Demographics

	Adult	Paediatric	Ancestral (Paediatric subset)	Omicron (Paediatric subset)
**N**	40	68	54	14
**Age**				
age min	22	0.78	0.91	0.78
age max	59	3.96	3.96	3.59
age median	27.4	2.24	2.37	2.06
**Sex**				
M	15	43	35	8
F	25	25	19	6
**Race**				
White	28	56	44	12
Black	3	9	8	1
Asian	9	11	9	2
**Ethnicity**				
NHL	39	55	47	8
HL	1	13	7	6

Table S2. Key Resources Table

Reagent	Source	Catalog Number
**Antibodies**		
BV421 anti-CD19	Biolegend	Cat#302233
BUV661 anti-CD38	BD Biosciences	Cat#612969
AF700 anti-IgG	BD Biosciences	Cat#561296
FITC anti-CD3	Biolegend	Cat#300406
FITC anti-CD14	Biolegend	Cat#325604
FITC anti-CD16	Biolegend	Cat#360716
BB700 anti-IgD	BD Biosciences	Cat#566538
PE anti-IgA	Miltenyi	Cat#130-116-878
SULFO-TAG Anti-Human IgG Antibody	MSD	Cat#D21ADF-3
**Chemicals, peptides and recombinant proteins**		
SARS-CoV-2 Biotyinlated Full Length Spike (WT, XBB.1.16, XFG, BA.3.2)	In-house	
SARS-CoV-2 Biotyinlated Receptor Binding Domain (WT, XBB.1.5, BA.3.2)	In-house	
SARS-CoV-2 Biotyinlated N-Terminal Domain (WT, XBB.1.5)	In-house	
BV605 Streptavidin	Biolegend	Cat#405229
PE-Cy7 Streptavidin	Biolegend	Cat#405206
Ghost Dye 510 Viability Dye	Tonbo	Cat#13-0870-T100
